# Magnesium Sulfate and Sufentanil for Patient-Controlled Analgesia in Orthopedic Surgery

**DOI:** 10.5812/aapm.11334

**Published:** 2014-02-28

**Authors:** Abass Sedighinejad, Mohammad Haghighi, Bahram Naderi Nabi, Poupak Rahimzadeh, Ahmadreza Mirbolook, Mohsen Mardani-Kivi, Majid Nekufard, Gelareh Biazar

**Affiliations:** 1 Anesthesiology Research Center, Guilan University of Medical Sciences, Rasht, Iran; 2 Anesthesiology Department, Rasoul-e-Akram Medical Center, Iran University of Medical Sciences, Tehran, Iran; 3 Orthopedic Research Center, Guilan University of Medical Sciences, Rasht, Iran

**Keywords:** Sufentanil, Magnesium Sulphate

## Abstract

**Background:**

Postoperative analgesia is one of the concerns of anesthesiologists and patients. Systemic opioid administration is the gold standard in reducing the severe pain after the surgery but some side effects prevent the use of adequate dosage of opioids.

**Objectives:**

The aim of this study was to evaluate the result of adding magnesium sulphate to sufentanil in patient-controlled intravenous analgesia (PCIA) system.

**Patients and Methods:**

In this randomized clinical trial, 60 patients candidate for lower limb orthopedic surgery were recruited in Poursina Medical Center for six months. They were randomly classified in two group of patient-controlled intravenous analgesia for postoperative pain control, one was group S [(sufentanil) (n = 30)] and the other was group S + M [(magnesium sulphate/sufentanil) (n = 30)]. The drug infusion rate was 5 mL/h. Each mL of solution in group S contained 1 mcg of sufentanil and in group M + S, 1 mcg of sufentanil and 200 mcg magnesium sulphate, respectively. Pain score, sedation score, satisfaction score, nausea and vomiting score were evaluated 6, 12, 24, 36 and 48 hours after surgery.

**Results:**

The demographic data between two groups were not significantly different. The pain scores after 6, 12 and 24 hours in S and S + M groups were significantly different. But the comparison of Visual Analogue Scale (VAS) scores after 36 and 48 hours didn’t show significant differences (P < 0.001). Comparison of the sedation, nausea and vomiting scores between two groups did not show any difference. But the number of patient’s satisfaction in S + M group was more than S group which suggests significant differences (P < 0.05).

**Conclusions:**

This study showed that magnesium sulphate added to sufentanil through PCIA is an effective method to alleviate pain in patients undergoing lower limb orthopedic surgery. Moreover, we found fewer side effects on magnesium-sufentanil regimen in terms of in nausea, vomiting, and sedation; and patients’ satisfaction in this regimen was more rather than that in the regiment of sufentanil alone.

## 1. Background

The optimal pain control after the major surgery is a concern for the Anesthesiologists and patients. Also, it should be noted that every treatment for pain control has some adverse drug effects, especially when the opioid is used (1-4). The patient controlled analgesia has been employed in clinical ward to get better post-operative pain control. Magnesium (Mg) is the fourth most prevalent cation in the body and activates approximately 300 enzymes systems; those involved in energy metabolism and nucleic acid synthesis. Magnesium is important in anesthesia practice for several reasons. First, the ion is essential for many biochemical reactions and its deficiency may cause clinical consequences during anesthesia or in intensive care unit. Second, the extensive use of magnesium sulphate in obstetric practice makes the anesthesiologists familiar with the pharmacological action of this drug and its interaction with anesthetic agents. Third, it’s some properties may be valuable in certain areas of anesthetic practice (5-7). As mentioned above, we need to use some methods or drugs to decrease the dosage of opioid (opioid sparing effect). So, in this study, we added magnesium sulfate , as an adjuvant drug, to sufentanil to relieve post-operative pain with the least side effects (6-9). There are many studies describing the analgesic effect of magnesium sulfate (8, 10). In fact, magnesium is proposed as an NMDA receptor antagonist that is used for the treatment of severe illnesses such as post-operative pain control, hypokalemia, premature labor and myocardial ischemic protection (9, 11). This study was planned to assess the effect of adding magnesium to sufentanil in PCA method to get better post-operative pain control in patients undergoing general anesthesia for lower limb orthopedic operation.

## 2. Objectives

Actually, various studies have been done regarding the role of magnesium sulphate in post-operative analgesia. Because there are some conflicting evidences to support analgesic efficacy of magnesium sulphate, it was attempted to study the role of magnesium sulphate for post-operative analgesia. The effect of magnesium plus sufentanil and sufentanil alone were compared regarding the Visual Analogue Scale (VAS), ramsay score, nausea and vomiting score and pethedine consumption. The findings of this study showed the beneficial effect of magnesium sulfate for effective pain control in post-operative period.

## 3. Patients and Methods

The agreement of the study was confirmed by the Ethical Committee of Anesthesiology Department, Guilan University of Medical Sciences. 

In a double-blind randomized and placebo-controlled clinical trial, 60 patients of those who were admitted for orthopedic surgery during the six months in Poursina hospital, included in this study. P value of 0.05 and a power of 80 % were considered based on the study of Kiran and Evans (12, 13). After obtaining the informed consent, the patients were assigned randomly in two different groups. Inclusion criteria were adult patients who scheduled for elective lower limb orthopedic surgery, aged between 20-60 years and classified into ASA 1 and 2. The patients who refused to fill out the informed consent or had any history of addiction, cardiac arrhythmia or renal disease were excluded from the study. Besides, if the operation took more than 90 minutes, the patient was excluded from study. Then, the patients were randomly classified into two groups, according to the table of random number. A resident of anesthesiology visited the patients before the operation and described the VAS for them. Meanwhile, he prescribed the premedication and 8 hours NPO for the patients. All of the patients were anesthetized by an anesthesiologist who was blinded to study and didn't participate in data collection. Furthermore, the anesthesiology resident who collected post-operative data were blinded to study and this process continued to end of the study. All the patients were fully informed about the study and blinded to their groups. Spinal anesthesia was done on all patients and each patient received 700 mL of normal saline over 15 min .Then , under sterile situation, 25 g whitacre spinal needle was inserted through L4-L5 intervertebral space, spinal blockade was performed by 100 mg (2 mL) of sterile, Lidocaine 5 % was injected regularly, and an appropriate sensory block level (T6-T7) was checked. Patients were divided in two groups; control and drug group that is S (sufentanil) and S + M (sufentanil + MgSO_4_), respectively. A patient - controlled intravenous pump (270 mL) (Chang hi China factory, China) was installed in both groups with an infusion rate of 5 mL /h. In control group, the PCA pump contains 40 mL sufentanil (200 mcg; Janssen-Cilag company, Belgium) plus 230 mL of normal saline whereas in drug group (S + M), the tank contains 40 mL sufentanil with 2000 mg MgSO_4_ 20 % (Samen Factory, Mashhad) with a total volume of 270 mL. Patients were evaluated at 6, 12, 24, 36 and 48 hours after filling the pump. Need to mention that all patients who transferred to recovery room were administered with PCA pump. So, the magnesium sulfate was infused through the PCA pump over 24 hours at the end of the operation in recovery room, without subsequent infusion. As mention above, the operation usually takes less than 90 min. The patient's primary states were pain score, nausea and vomiting, restfulness; and satisfaction were recorded in questioner. The pain score was estimated based on visual analog scale from zero (pain free) to 10 (maximum level of pain). Nausea and vomiting scored from 1 to 4 (1 = without nausea and vomiting, 2 = nausea without vomiting, 3 = less than two times vomiting, 4 = severe vomiting more than two times) and satisfaction scored from 1 to 4 (1 = low, 2 = intermediate, 3 = good, 4 = excellent) (14, 15). The restlessness score was estimated based on Ramsay criteria from 1 to 6 scoring system (16). The patients with pain scores more than 3 were treated with 30 mg of intravenous Mepridine and number of injections were recorded. This dosage of Mepridine could be repeated per 30 minutes and after each pethidine administration, the side effects were monitored. All of these data were registered in questioner paper. The data were analyzed using SPSS 16. The quantitative data such as mean and standard deviation and two qualitative data such as frequency rate and percentage have been shown in all cases, P < 0.05 was considered statistically significant.

## 4. Results

**Figure 1.**
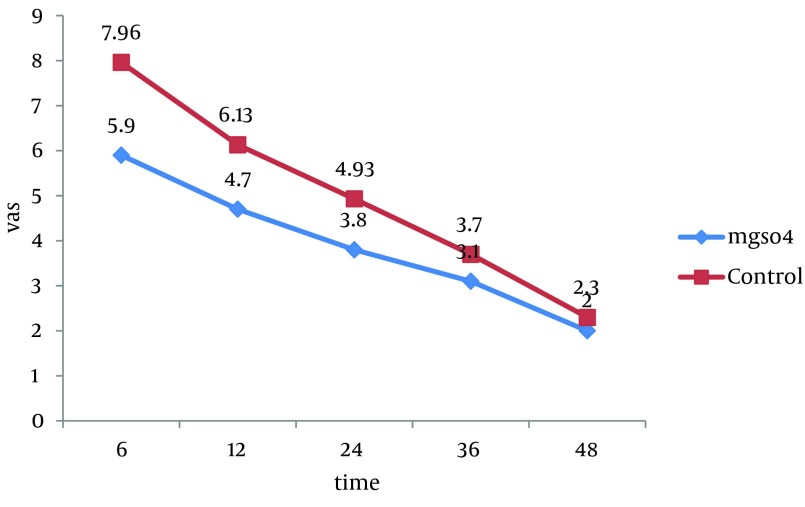
The Comparison of Nausea and Vomiting Mean Number in Two Groups at Different Time

**Figure 2.**
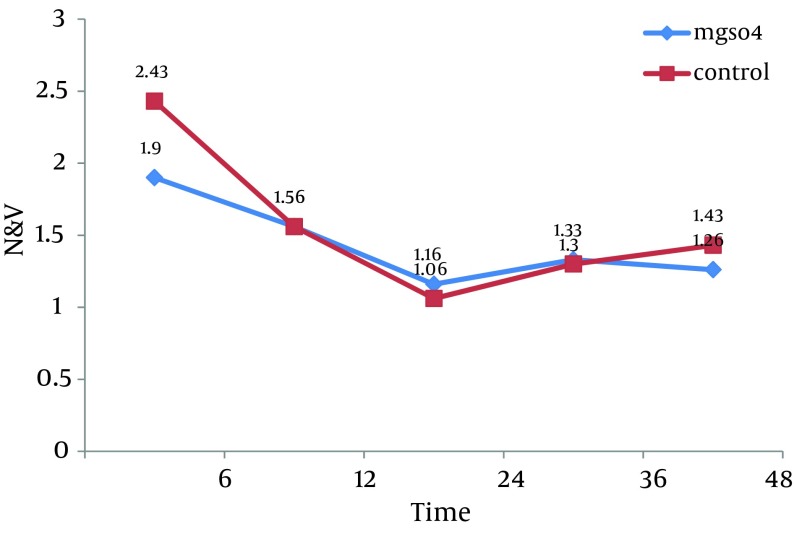
The Comparison VAS Score Between Two Groups in Different Time of Study

All 60 patients divided into two groups (30 patients in each group).There were no significant differences between these groups regarding the demographic data like age, weight, sex and body mass index (BMI). Almost all of patients in each group were men (Table 1). Visual Analogue Scale (VAS) was evaluated 6, 12, 24, 36 and 48 hours after the surgery and compared in both groups. Pain scores decreased significantly in magnesium sulfate groups in the first 24 hours, but no significant differences were seen in the 36 and 48 hours. The general linear model and repeated measurement showed a significant difference between VAS score 6, 12, and 24 hours after the surgery (P < 0.0001) (Table 2, Figure 1). Comparison of sedation scores suggested that there were no significant differences in both groups at all the times of trial. The general linear model and repeated measurement didn't show a significant difference between Ramsay score at different times (P = 0.922) (Table 3). The nausea and vomiting scores showed in Table 2 were similar in both groups. The general linear model and repeated measurement didn’t show a significant difference between nausea and vomiting score at different times after surgery (Table 3, Figure 2). The patient’s satisfaction rates were higher in magnesium sulfate group than control (Table 2). This result from the chi-square test showed a significant correlation between two groups. Finally, based on t-test examination, it was found that pethidine consumption, regarding the number of injection, was higher in control group (P = 0.0001) than in Magnesium Sulfate group which is presented in Table 2 (3.73 ± 1.28 vs. 1.7 ± 0.7, respectively).

**Table 1.** Demographic Characteristics of Patients in Two Magnesium Sulfate and Control Groups

	Mg SO_4_	Control Group	P value
**Gender, No. %**			
Men	22 (73.3)	20 (66.7)	0.799 ^a^
Women	8 (26.7)	10 (33.3)	0.799 ^a^
**Age, Mean ± SD, y**	35 **± **13.92	39.46 **± **12.95	0.25^b^
**BMI **^**c**^**, No. %**			
≤ 19	0 (0)	1 (33)	0.69^d^
19-25	9 (30)	7 (23.3)	0.69
25-30	15 (50)	17 (56.7)	0.69
≥ 30	6 (20)	5 (16.7)	0.69

^a^ Fisher exact test.

^b^ T-test.

^c^ Abbreviations: BMI, body mass index

^d^ Chi square test.

**Table 2.** The Comparison of VAS in Different Time Between Two Groups

VAS ^a^, h	MgSO_4_, Mean ± SD	Control Group, Mean ± SD	P value
**6**	5.9 **± **1.2	7.96 **±** 1.12	0.0001, F = 7.1
**12**	4.7 **± **1.08	6.13 **±** 1.13	0.0001, F = 4.99
**24**	3.86 **±** 1.5	4.93 **±** 1.25	0.0004, F = 2.98
**36**	3.1 **±** 1.21	3.7 **± **1.31	0.072, F = 1.83
**48**	2.3 **± **1.05	2 **±** 1.14	0.296, F = 0.05

^a^ Abbreviations: VAS, visual analog score

**Table 3.** The Comparison of Ramsay Score and N&V Score Between Two Groups

	MgSO_4_	Control Group	P value
**Ramsay Score, Mean ± SD, hr**			
6	2 ± 0.74	2 ± 0.64	0.922, F = 0.23
12	1.73 ± 0.73	1.83 ± 0.83	0.922, F = 0.23
24	1.6 ± 0.56	1.53 ± 0.57	0.922, F = 0.23
36	1.66 ± 0.6	1.73 ± 0.63	0.922, F = 0.23
48	1.6 ± 0.62	1.5 ± 0.57	0.922, F = 0.23
**Patient’s Satisfaction, No. %**			
Poor	3 (10)	11 (36.7)	0.0001^a^, df = 3
Moderate	5 (16.7)	13 (43.3)	0.0001^a^, df = 3
Good	13 (43.3)	6 (20)	0.0001, df = 3
Excellent	9 (30)	0	0.0001, df = 3
**Nausea and Vomiting, mean ± SD, hr**			
6	1.9 **±** 1.06	2.43 **±** 1.16	0.069^b^, F = 1.85
12	1.56 **±** 0.77	1.56 **±** 0.72	1 ^b^, F = 0.0
24	1.16 **±** 0.46	1.06 **±** 0.25	0.3 ^b^ 02, F = 1.05
36	1.33 **±** 0.56	1.3 **±** 0.46	0.8 ^b^, F = 0.254
48	1.26 **±** 0.44	1.43 **±** 0.5	0.182^b^, F = 1.35
**Pethidine Consumption, No of Injection**	1.7 (0.7)	3.73 (1.28)	0.0001^b^, F = 7.6

^a^ Chi square test.

^b^ Pair test.

## 5. Discussion

This study revealed that addition of sufentanil to magnesium sulfate can reduce the post-operative pain significantly and pain reduction after passing 24 hours of operation is considerable. The mechanism of analgesic effect of magnesium is not clear but may interfere with calcium channel and NMDA receptors may play a role. Magnesium acts as a noncompetitive inhibitor of the inositol 1, 4, 5-triphosphate gated calcium channel and IP3 binding (9, 17). The analgesic action of some calcium channel blocker could be mediated by an increase of the nociceptive threshold resulting from interference with calcium influx because the latter is important for the release of neurotransmitters and other substances (18-20).

The patient controlled analgesia system is a highly satisfactory method to reduce the post-operative pain (1, 21-24). In this manner, the results showed that patients are generally satisfied with PCA system that had been a qualified pain control with adjuvant combination of magnesium sulfate to sufentanil PCA (21). It showed the improvement of pain control following general surgical procedures (10, 20, 25). The pain assessment was the primary postoperative outcome as defined by visual analogue score (2, 3, 26) . Secondary clinical outcome included postoperative nausea and vomiting. Sedation also was assessed by Ramsay scoring system. The nausea and vomiting was similar in these two groups. The presence of nausea and/or vomiting was assessed at several times after the operation. The sedation score (Ramsay) in both groups was similar but patient’s satisfaction score in magnesium sulfate group was interestingly higher. hypothetically, magnesium group consumed the pethidine less than sufentanil group. As mentioned above, some chronic pain syndrome can be treated via intravenous or intramuscular opioids administration (4, 13). Regarding some adverse effect of opioids administration such as nausea, vomiting, respiratory depression and constipation, hospitalization can be prolonged. Patients using PCA were recovered compared to those using conventional analgesia, without any side effects (25, 27) but, it is costly. In this present study, the aim was not only to evaluate magnesium as an anesthetic agent but also to find out whether it could decrease opioids requirement with less adverse effect. This dosage basically, has been reported to be safe without any adverse effect such as nausea, vomiting and over sedation. It was noted that patients’ satisfaction in drug group showed the supreme analgesia of magnesium sulfate, meanwhile one of the limitations of our study which affected on data analysis was unmeasured data during first six hours, which seems good to evaluate the VAS, nausea and vomiting, and ramsay score before that time. We demonstrated some probability mechanism for nociceptive action of magnesium but further studies about the interaction between magnesium and opioids (Sufentanil) are needed. Our data showed a significant reduction in VAS score and pethedine consumption between two groups that is compatible with the results of several studies (17, 19). The effect of magnesium on preoperative analgesic requirements was first evaluated by Koining et al. Based on their data, magnesium can be a useful adjuvant for preoperative analgesic management by reducing fentanyl requirement (5). In the other study, it was concluded that if magnesium is used as a bolus after induction, it can reduce the anesthesia requirement significantly (9). Unfortunately, we didn't measure the concentration of serum magnesium. The dose-response curve for magnesium regarding its synergistic effect during pain control should be evaluated in further studies. Fast rehabilitation after orthopedic surgery was the aim of this research.

Pain control is a multi-modal approach (6, 7, 22), so all the studies and all pharmacologic interventions try to find better ways to for pain control. Our study had some limitations, but generally showed that attenuation of post-operative pain through magnesium added to opioids suggests a new pharmacologic therapy to control better post-operative pain.
